# A new three-locus model for rootstock-induced dwarfing in apple revealed by genetic mapping of root bark percentage

**DOI:** 10.1093/jxb/erw001

**Published:** 2016-01-29

**Authors:** Nicola Harrison, Richard J. Harrison, Nuria Barber-Perez, Emma Cascant-Lopez, Magdalena Cobo-Medina, Marzena Lipska, Rebeca Conde-Ruíz, Philip Brain, Peter J. Gregory, Felicidad Fernández-Fernández

**Affiliations:** ^1^East Malling Research, New Road, East Malling, Kent ME19 6BJ, UK; ^2^Centre for Food Security, School of Agriculture, Policy and Development, University of Reading, Whiteknights, PO Box 237, Reading RG6 6AR, UK

**Keywords:** Apple, grafting, *Malus × domestica*, root anatomy, root bark percentage, root cortex, rootstock, rootstock-induced dwarfing, scion.

## Abstract

Rootstock-induced dwarfing of apple scions revolutionized global apple production during the twentieth century, leading to the development of modern intensive orchards. A high root bark percentage (the percentage of the whole root area constituted by root cortex) has previously been associated with rootstock-induced dwarfing in apple. In this study, the root bark percentage was measured in a full-sib family of ungrafted apple rootstocks and found to be under the control of three loci. Two quantitative trait loci (QTLs) for root bark percentage were found to co-localize to the same genomic regions on chromosome 5 and chromosome 11 previously identified as controlling dwarfing, *Dw1* and *Dw2*, respectively. A third QTL was identified on chromosome 13 in a region that has not been previously associated with dwarfing. The development of closely linked sequence-tagged site markers improved the resolution of allelic classes, thereby allowing the detection of dominance and epistatic interactions between loci, with high root bark percentage only occurring in specific allelic combinations. In addition, we report a significant negative correlation between root bark percentage and stem diameter (an indicator of tree vigour), measured on a clonally propagated grafted subset of the mapping population. The demonstrated link between root bark percentage and rootstock-induced dwarfing of the scion leads us to propose a three-locus model that is able to explain levels of dwarfing from the dwarf ‘M.27’ to the semi-invigorating rootstock ‘M.116’. Moreover, we suggest that the QTL on chromosome 13 (*Rb3*) might be analogous to a third dwarfing QTL, *Dw3*, which has not previously been identified.

## Highlight

Identification of three quantitative trait loci associated with root bark percentage in apple rootstocks is predictive of rootstock-induced dwarfing of the scion.

## Introduction

The technique of grafting has been practised for hundreds of years in order to maintain, crop and propagate many different fruits (Feree and Carlson, 1987). Grafted trees are typically composed of two parts, the rootstock and the scion. The rootstock contributes the root system and the base of the stem while the scion is grafted on top of the rootstock and is the part of the tree that produces the fruit. The point of grafting between the two plant parts is known as the graft union (Mudge *et al.*, 2009). All temperate tree fruit crops grown commercially throughout the developed world are grafted onto rootstocks selected for specific traits such as growth control and early fruit production. Growth-controlling rootstocks have enabled the transition of low-yielding traditional orchards to high-density modern fruiting orchards, mainly through the vigour control they impart to the scion to produce compact trees (Mudge *et al.*, 2009). This vigour control is gradual and cumulative, and interactions between rootstock, environment and scion can introduce variation into the growth of the composite tree. Rootstocks can also influence precocity and yield efficiency in addition to controlling some diseases (Rom and Carlson, 1987). Between 1913 and 1915, researchers at the Wye College Fruit Experiment Station at East Malling collected a range of clonally propagated apple rootstocks used by nurseries in the UK and continental Europe and embarked on a process of detailed phenotypic characterization. This led to the description of the first nine ‘Malling’ series of apple rootstocks (‘M.1’ to ‘M.9’), which included both dwarfing (‘M.8’ and ‘M.9’) and non-dwarfing types (‘M.1’ and ‘M.2’; Hatton, 1917; Hatton, 1918). Breeding programmes in Europe, North America and New Zealand have since used the ‘Malling’ series to develop novel apple rootstocks with improved pest and disease resistance that are better adapted to their local conditions (Cummins and Aldwinckle, 1974; Webster *et al.*, 2000). Most commercially available dwarfing apple rootstocks can be traced to just one or two original sources, namely ‘M.9’ and ‘M.8’ (Russo *et al.*, 2007), which have been found to be closely related to *Malus × domestica* using molecular analysis (Oraguzie *et al.*, 2005; Fazio *et al.*, 2011). A practical problem when using invigorating genotypes as a source of pest or disease resistance in breeding programmes is that the dwarfing effect of one parent is frequently lost in the first generation. As a result of this, dwarfing genotypes only reappear when F1 genotypes (derived from a dwarfing and a non-dwarfing parent) are crossed again to another dwarfing genotype. This is a serious limitation for breeding programmes because of the long generation cycles required to confidently evaluate rootstock performance and its effect on the scion. Robust molecular markers strongly linked to rootstock-induced dwarfing are a much-needed tool to hasten rootstock breeding.

Assessment of vigour control by a rootstock is not easy and earlier work to determine the genetic basis of vigour control in apple rootstocks has focused on above-ground traits such as trunk cross-sectional area, or visual assessment of growth habit and internode length (Seleznyova *et al.*, 2003; Rusholme-Pilcher *et al.*, 2008).

Previous studies by Rusholme-Pilcher *et al.* (2008) and Fazio *et al.* (2014) identified two quantitative trait loci (QTLs) associated with rootstock-induced dwarfing: *Dwarfing 1* (*Dw1*), at the top of linkage group/chromosome 5 (LG05) between markers CH03a09 and NZraAM18-700, and *Dwarfing 2* (*Dw2*), on linkage group/chromosome 11 (LG11) between markers CH02d08 and C13243 (Rusholme-Pilcher *et al.*, 2008; Fazio *et al.*, 2014). The second QTL, *Dw2*, was initially identified assuming an additive model with *Dw1*, but was proposed to have non-additive effects after further analysis. The genetic model put forward by Fazio *et al.* (2014) was more predictive where either of the dwarfing loci was homozygous, and gave less accurate predictions of phenotype where either or both loci were heterozygous (Fazio *et al.*, 2014). A recent study by Foster *et al.* (2015) identified *Dw2* as an additive QTL, and they suggested that ‘*Dw1* has a stronger effect on rootstock-induced dwarfing than *Dw2*, and that *Dw2* may act as an enhancer of *Dw1*’ (Foster *et al.*, 2015). Foster *et al* found similar results to Fazio *et al*., with their model only able to account for 68.6% of the observed variation. Both studies reported cases where *Dw1* was present yet the expected phenotype did not behave as predicted.

While many previous studies on dwarfing apple rootstocks have concentrated upon measuring a secondary conferred trait (i.e. the manifestation of vigour control in the scion), this study focuses on a primary rootstock trait known as root bark percentage. A high proportion of root cortical cells (hereafter termed root bark percentage) in the root of an apple rootstock has previously been associated with the ability of the rootstock to reduce the vigour of a grafted scion (Beakbane and Thompson, 1947). The aim of the present study was to identify QTLs for root bark percentage and to confirm whether there was an association with rootstock-induced dwarfing in the M432 mapping population. We used the M432 apple rootstock mapping population together with its associated genetic map (Fernández-Fernández *et al.*, 2012; Antanaviciute *et al.*, 2012) to identify QTLs associated with root bark percentage. We also present a model of rootstock-induced dwarfing, describing the nature of allelic variation and how this determines the ability of the rootstock to control vigour.

## Materials and methods

### Plant material

#### M432 field population

The M432 apple rootstock mapping progeny used in this study has been previously reported (Fernandez-Fernandez *et al.*, 2012). It is a subset of a larger seedling population raised in 2004 from a cross between the dwarfing rootstock ‘M.27’ (‘M.13’ × ‘M.9’) and a semi-vigorous rootstock ‘M.116’ (‘M.M.106’ × ‘M.27’). The M432 family originally comprised 257 individuals of which 120 (population A) were maintained as low hedges (ungrafted and cut hard to encourage shoot production) for 10 growing seasons. The remaining individuals were budded with a columnar scion (East Malling Research (EMR) selection number SA544-28) in 2005 (population B). From the latter population, 68 (population B1) were cut back below the graft union in December 2007 and treated as population A until they were all grubbed (removal of trees from a field planting) in December 2014. The rest of the seedlings (population B2) were evaluated as part of the breeding programme for their effect on the scion and lifted in 2015 for propagation. The mapping progeny comprised a total of 140 seedlings (89 and 51 from populations A and B1, respectively), DNA from which was used to develop a combined simple sequence repeat (SSR) linkage map (Fernández-Fernández *et al.*, 2012) and single nucleotide polymorphism (SNP)-based maps of the parental genomes (Fernández-Fernández *et al.*, 2012; Antanaviciute *et al.*, 2012; Troggio *et al.*, 2013).

#### Reference rootstock cultivars in the field

Three replicates each of four commercially available rootstock cultivars (‘M.9’, ‘M.26’ ‘M.27’ and ‘M.M.106’) were interspaced in the planting of the M432 progeny and were treated like population B1.

#### Grafted subset of M432 mapping population

In December 2010, four or more replicates of each of the seedlings in the M432 mapping population (populations A and B1) were propagated through hardwood cuttings. Successfully rooted cuttings were grafted with ‘Royal Gala’ in January 2012 and grown in pots outdoors for three growing seasons. Replicates of the parental genotypes (‘M.27’ and ‘M.116’) were also grafted and grown in pots alongside the seedlings. In January 2015, trees from 37 seedlings (for which five or more replicates grafted with ‘Royal Gala’ were available) as well as the parents were measured for rootstock-induced dwarfing traits (e.g. stem diameter).

### Determination of root bark percentage

#### M432 field population

In 2014, the 122 seedlings of the M432 mapping population that survived the 10 growing seasons were lifted from the field. Twelve root segments (2–8mm in diameter, 100–120mm in length) were excised from each root system using secateurs, placed into a labelled polythene bag with moist tissue to prevent desiccation of the roots and stored at 4 °C before analysis. The roots were then carefully washed using a nailbrush to remove all the soil. For each root segment, a scalpel or a utility knife was used to remove a ring of bark (cortex) approximately 2–3mm in length, leaving behind the stele of the root. Digital calipers were used to make pairs of measurements of the root with and without the bark. The cross sectional area of the root and the percentage of total area occupied by the root bark were calculated for each sample, assuming that the root section was a perfect cylinder. The percentage of root bark at a standard root diameter of 7.5mm was then inferred using regression analysis.

#### Grafted subset of M432 mapping population

The method used was as described above for the M432 field population but modified to allow for the small size of the young root systems. Three root segments (2–8mm in diameter) were sampled for each replicate and each root was measured twice with the second set of measurements taken at 90° to the first set; the two values were then averaged.

### Measurements of stem diameter in grafted trees

Stem diameter measurements were made on the grafted replicates of M432 seedlings and the parents ‘M.27’ and ‘M.116’ to provide an indication of the degree of dwarfing of the genotypes. Measurements were taken 20cm above the graft union on all trees. This height was chosen to be clear of the lowest branch thereby preventing any measurement distortion. Digital calipers were used to measure each trunk twice, with the second set of measurements taken at a 90° angle to the first. Means were calculated and used for subsequent analyses.

### Root microscopy and staining

Roots were collected from apple rootstocks, washed free of soil and fixed in FTT fixative (4% (w/v) formaldehyde with 0.1% Tween-20 and 0.1% Triton X-100). Transverse sections (20–25 μm) were obtained using a sliding microtome (Reichert ‘Om E’), placed onto a microscope slide and stained with 0.05% aniline blue (w/v) in 0.067M phosphate buffer at pH 8.5. The stain was not rinsed. Sections were covered with a glass coverslip before imaging with a Leica DMI6000 fluorescence microscope using two filters: A4 (green, ex: 340–380nm; em: 450–490nm) and L5 (red, ex: 460–500nm; em: 512–542nm). The resulting images were overlaid to produce the final image, which was digitally captured using a Leica DFC450C camera with the following settings: exposure: 800–2; intensity: 4.

### Genome sequencing and assembly

High quality DNA was extracted from the apple rootstocks ‘M.9’, ‘M.27’, and ‘M.116’ using the Qiagen DNeasy plant tissue kit following the standard protocol (Qiagen, UK). The Genome Analysis Centre (TGAC), UK, performed DNA library preparation for 100bp paired end (PE) read sequencing using standard Illumina chemistry and sequenced on an Illumina HiSeq 2000 generating a minimum of ×50 coverage. The average insert size of the libraries was 621bp. Low quality reads were removed using fastq-mcf (Aronesty, 2013). The sequence reads were aligned to the reference apple genome: ‘Golden Delicious’ *Malus × domestica* v1.0 pseudo haplotype downloaded from the Genome Database for Rosaceae (GDR) (Jung *et al.*, 2008; Velasco *et al.*, 2010) using reference-guided assembly (RGA). The commercial software Geneious® was used for RGA, multiple chromosome alignment and data visualization (www.geneious.com).

### QTL mapping

Histograms of root bark percentage were visualized using R and a test for normality (QQ-plot) was carried out. Raw data (*W*=0.9648, *P*<0.006) were non-normally distributed, and log-transformed data (*W*=0.979, *P*<0.08) were on the boundary of significance. Log-transformed values were subsequently used for QTL analysis. Exploratory QTL mapping was carried out with Kruskal–Wallis (K-W) non-parametric ANOVA on the combined map of ‘M.27’ and ‘M.116’. The K-W ANOVA approach allows both the identification of QTLs specific to one parent and QTLs that are present in both parental genotypes to be estimated, rather than carrying out separate QTL analysis on the two parental linkage maps. K-W analysis was used as an exploratory data analysis tool to identify main QTL effects. Because it was found during this work that there was significant non-additivity between main effect QTL, interval mapping (which assumes an additive model of genetic effects) was not used (Lark *et al.*, 1995). The K-W QTL analysis was carried out in the MapQTL5 software package (Kyazma, Wageningen, The Netherlands), using the map published by Antanaviciute *et al.* (2012).

### Marker development

The highest scoring SNP marker for each QTL was used to select the chromosomal regions for marker development. Microsatellite markers were manually designed, using the draft rootstock whole genome alignment (N. Harrison, unpublished data), in regions where significant SNPs from the QTL mapping were located. Using the rootstock whole genome alignment for chromosomes 5, 11 and 13, three primer pairs were designed around simple sequence repeats and indel features, and screened on the M432 mapping population for segregation. The primer sequences are given as follows with primer name followed by the 5′–3′ DNA sequence:

EM_Rb1_F gcgttgaaggaggttatcgag; EM_Rb1_R acatctatatcattc aagtac; EM_Rb2_F gagctatagaggctggattag; EM_Rb2_R gcagacttgctccaggtaac; EM_Rb3_F gaggcaatctaaataatgaag; EM_Rb3_R caagcacactgccttggtcaac.

### Sequence-tagged site marker analysis

Primer pairs were labelled on the forward primer with 6-FAM fluorescent dye (IDT, Belgium) using an M13-tailed primer in a two-step reaction as described by Schuelke (2000). PCR reactions for sequence-tagged site (STS) markers were performed using the ‘Type-it’ PCR mastermix (Qiagen) following the manufacturer’s recommendations, in a final volume of 12.5 μl. PCR reactions were carried out using the following PCR cycles: an initial denaturation step of 94 °C for 5min was followed by 10 cycles of 94 **°**C for 30s, an annealing temperature of 55 **°**C decreasing by 0.5 **°**C per cycle until 50 °C for 45s and 72 °C for 60s, followed by 25 cycles of 94 °C for 30s, 50 °C for 45s and 72 °C for 60s with a final extension step at 72 °C for 15min. PCR products were fractionated by capillary electrophoresis through a 3130 Genetic Analyzer (Applied Biosystems). Data generated were collected and analysed using the GENESCAN and GENOTYPER (Applied Biosystems) software.

### Statistical analysis

Statistical analyses were performed using the statistical software package ‘R’ version 3.1.0 using the lm function (R Core Development Team, 2008) and GenStat for Windows 14th Edition (VSN International). The models presented were developed by sequentially adding fixed effects to the regression analysis, beginning with single additive factors (chr 5, chr 11, chr 13), followed by interaction terms. Final models were selected, based upon significant terms in the model selection.

## Results

### Root bark percentage

Root bark percentage was examined in the field-grown, ungrafted M432 rootstock population (populations A and B1) and found to vary in a genotype-dependent manner. Field controls included ‘M.27’, ‘M.9’, ‘M.26’ and ‘M.M.106’ (Table 1); unfortunately, liners—young rootstocks, typically 1 or 2 years old—of ‘M.116’ were not available for planting when the field plot was established and it is not, therefore, included in Table 1. (Liners can be produced by hardwood cuttings or micro-propagation or lifted from a stool bed as a single stem rooted trunk; they can be bench grafted in the winter and then planted out in the field or pots or ‘lined’ in a nursery field for summer budding.) Table 1 shows that dwarfing rootstocks had a higher percentage of root bark than semi-invigorating rootstocks. It is noteworthy that ‘M.27’, which has been characterized as more dwarfing than ‘M.9’, has a slightly lower root bark percentage than its parent, ‘M.9’ in this study.

**Table 1.** Root bark percentage in common field-grown apple rootstocks, measured after 10 growing seasons

Rootstock	Average root bark percentage (at 7.5mm)	SEM	Vigour
M27	72.92	2.00	Strongly dwarfing
M9	76.89	0.76	Dwarfing
M26	64.05	2.13	Semi-dwarfing
MM106	52.56	4.31	Semi-invigorating

SEM: standard error of the mean.

**Fig. 1.**
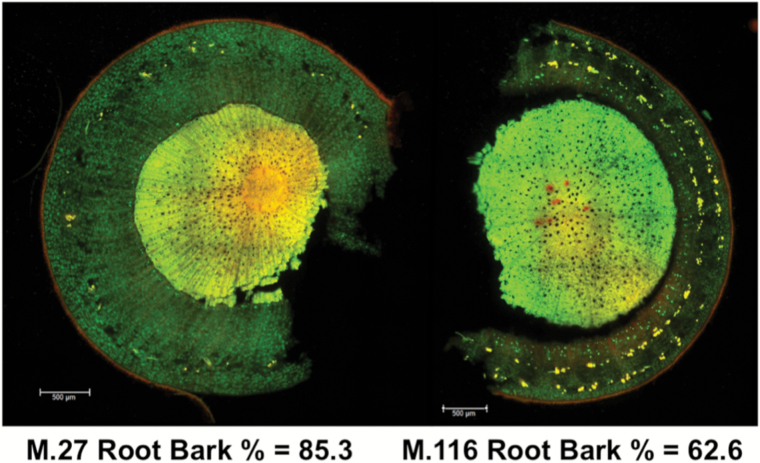
Root bark microscopy images depicting the root bark percentages for the two parents of the M432 mapping population, ‘M.27’ and ‘M.116’.

Root bark percentage was measured for both ‘M.27’ and ‘M.116’ using pot-grown rootstocks grafted with ‘Royal Gala’ and grown for 3 years. The dwarfing rootstock ‘M.27’ had a root bark percentage of 85.3%, while the semi-invigorating rootstock ‘M.116’ had a smaller root bark percentage of 62.6%. This phenotypic difference was clear when transverse sections of root were stained and visualized under a microscope (Fig. 1). While ‘M.116’ had a slightly higher root bark percentage than its semi-invigorating parent ‘M.M.106’ (52.5%, Table 1), it was still significantly different from its dwarfing parent ‘M.27’ (Fig. 1 and Table 1). These preliminary data agree with expectations derived from much earlier studies (Beakbane and Thompson, 1947).

### Variation in root bark percentage

**Fig. 2.**
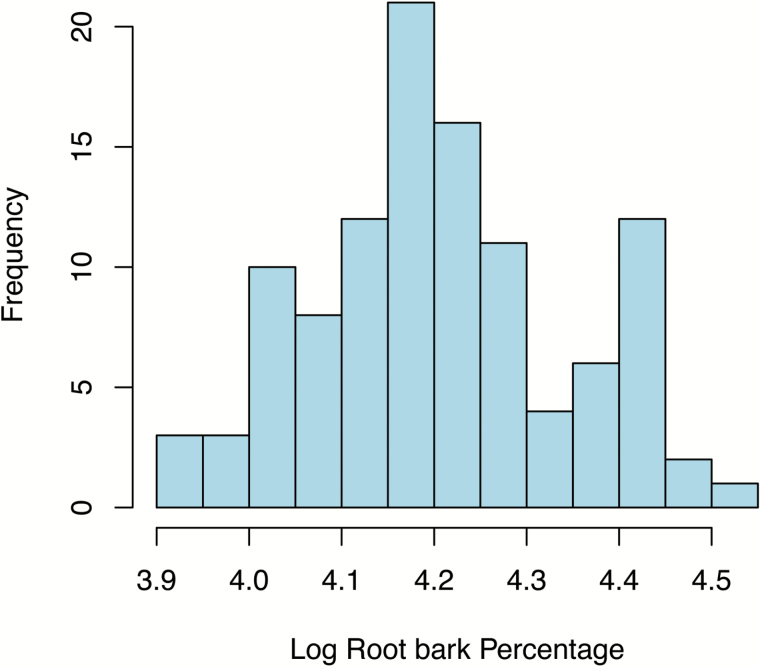
Histogram of phenotypic distribution of log-transformed root bark percentage data for the M432 mapping population.

The log-transformed distribution of root bark percentages for the 122 individuals of the M432 mapping population had a unimodal distribution pattern with clear segregation (Fig. 2). Log-transformed values were normally distributed and were used for subsequent QTL mapping of loci involved in modulating root bark percentage.

### Identification of QTLs

**Fig. 3.**
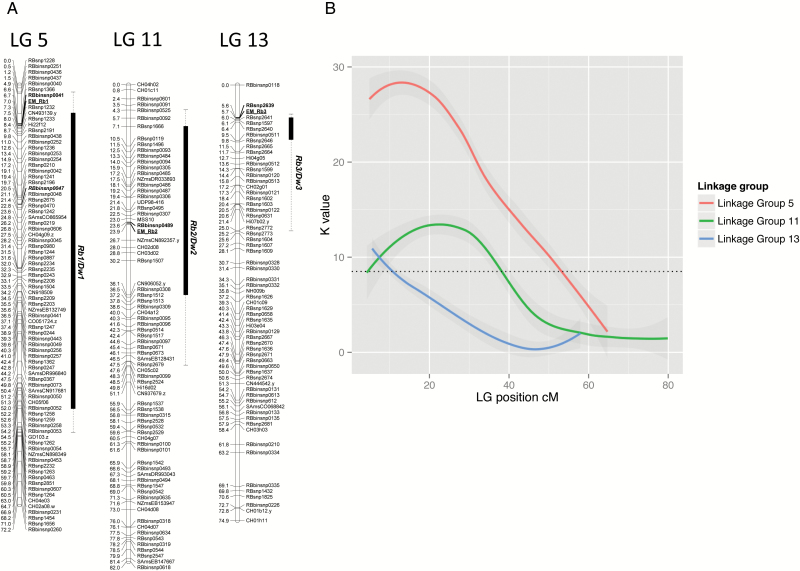
(A) QTLs by linkage group. Markers with a significance level of *P*<0.005 are highlighted in boxes, with *P* values between *P*>0.005 and *P*<0.05 shown as whiskers. (B) QTLs by position, including both SNP and STS marker data. *K* value of the QTL plotted by genetic distance in cM along linkage group 5 (red), linkage group 11 (green) and linkage group 13 (blue). The dotted line indicates the *K* value at the threshold for significance (*P*<0.05).

QTL mapping was carried out using K-W testing and revealed three loci involved in the determination of root bark percentage, located on chromosomes 5, 11 and 13 (Table 2 and Fig. 3A), labelled *Rb1(a*), *Rb2(b*) and *Rb3(c*), respectively. *Rb1(a*) was present only in ‘M.27’, while *Rb2(b*) and *Rb3(c*) were detected in both parental genotypes. All QTLs were highly significant (Table 2) with *Rb1(a*) and *Rb2(b*) showing clear maxima on their respective linkage groups (Fig. 3B).

**Table 2.** Most significant SNP markers associated with the QTLs *Rb1, Rb2* and *Rb3*

Chr	Position (cM)	SNP	*K* statistic	Significance	Marker origin
5	20.47	RosBREEDSNP_SNP_AC_2429897_Lg5_00179_MAF40_1681882_exon1	32.1	*******	M27
11	23.637	RosBREEDSNP_SNP_CA_8702100_Lg11_00735_MAF40_1677605_exon4	13.409	****	M27 and M116
13	5.6	RosBREEDSNP_SNP_GT_2194655_Lg13_00098_MAF30_ MDP0000188704_exon3	12.648	****	M27 and M116

Chr: chromosome.

Significance levels: ****: 0.005; *******: 0.0001.

**Fig. 4.**
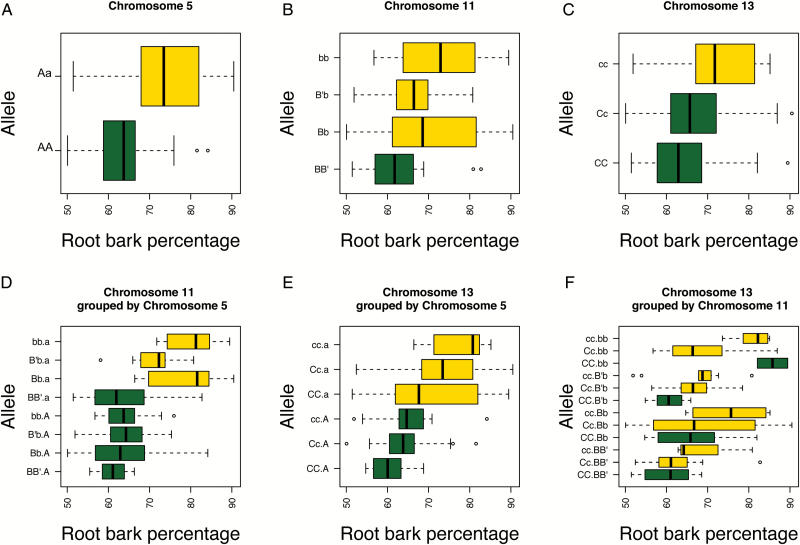
Boxplots depicting the effects of the rootstock genotype upon the expression of the root bark percentage trait. *Rb1* alleles on LG05 are depicted as (A) in repulsion to the QTL and (a) in coupling with the QTL; *Rb2* alleles on LG11 are depicted as (B) in repulsion to the QTL, ‘M.116’ allele, (B′) in repulsion to the QTL, ‘M.27’ allele, and (b) in coupling with the QTL; and *Rb3* alleles on LG13 are depicted as (C) in repulsion to the QTL, and (c) in coupling with the QTL. (A) the effects of *Rb1* when analysed alone. (B) The effects of *Rb2* alone. (C) The effects of *Rb3* alone. (D) pairwise comparisons of *Rb1* and *Rb2*, revealing significant non-additive effects between *Rb1* and *Rb2*, which vary depending upon the allelic combination at LG11. (E) This phenomenon is not observed for *Rb1* and *Rb3*, which appears to exert an effect independent of *Rb2*. (F) *Rb2* significantly affects the expression of *Rb3*, again dependent upon the allelic combination present at *Rb2.*

Plots of the progeny grouped by single SNP markers (STS markers in the case of QTL *Rb2* in order to resolve all parental haplotypes), reveal that, on average, each marker alone appears to have only a moderate effect on root bark percentage depending upon the allelic state (Fig. 4A–C). However, pairwise marker analysis revealed that there were substantial non-additive effects between *Rb1(a*) and *Rb2(b*) (Fig. 4D and Supplementary Fig. S1 at *JXB* online), but not *Rb1(a*) and *Rb3(c*) (Fig. 4E). These observations suggest a linear mixed model. A model that included an interaction between *Rb1(a*) and *Rb2(b*) was significant, but inclusion of interaction terms between *Rb1(a*) and *Rb3(c*), or *Rb2(b*) and *Rb3(c*) did not significantly improve this model (Supplementary Table S1). Specifically, the activity of *Rb1(a*) was dependent on the presence of *Rb2(b*) and vice versa (Fig. 4D and Supplementary Fig. S2). This is substantiated by the observation that the classes containing *Rb1(a*) but not *Rb2(b*), or *Rb2(b*) in the absence of *Rb1(a*) display phenotypes that are, on average, similar to the class lacking both *Rb1(a*) and *Rb2(b*) (Fig. 4D). The class of individuals with the highest root bark percentage in this pairwise comparison is the class containing *Rb1(a*) and two copies of *Rb2(b*) (one allele from ‘M.27’ and one from ‘M.116’). Alleles inherited from ‘M.27’ and ‘M.116’ in repulsion to the *Rb2(b*) allele, i.e alleles *Rb2(BB′*) in Fig. 4, appear to have different effects upon the ‘penetrance’ or expression of the root bark phenotype when present with the *Rb2(b*) allele (Fig. 4D). The *Rb2(B*) allele from ‘M.27’ appears to suppress the effects of *Rb2(b*) (though not to the level of the *Rb2*-lacking class), while the *Rb2(B′*) allele from ‘M.116’ increases the root bark percentage to levels comparable to the homozygous *Rb2(bb*) class, though with greater variance than the homozygous class.

Again, as seen with *Rb1(a*) when *Rb2(b*) was present (Fig. 4D), the expression of *Rb3(c*) appeared to be dependent on the combination of alleles at *Rb2*, with the *Rb2(B′*) allele apparently compensating for the homozygous state of *Rb3(cc*). In this pairwise analysis, it was clear that the allelic status of *Rb1* confounded the analysis of *Rb2* and *Rb3* due to small sample sizes of some genotypic classes. If there were no effect of *Rb2* in the absence of *Rb3*, the genotypic class ‘*Rb2(bb) Rb3(CC*)’ would predict a low root bark percentage. However, on examination of the allelic status of *Rb1* in this class, which comprised only two individuals, it was found that both individuals contained *Rb1(a*) and therefore had a high root bark percentage due to the interaction between *Rb1(a*) and *Rb2(b*).

To test whether the effect of *Rb3(c*) occurs regardless of the allelic status of *Rb1* (Fig. 4E) or *Rb2* (Fig. 4F), a three-way analysis of *Rb* loci was carried out. With *Rb2(BB′*), expression of *Rb3(c*) appeared to show only a pronounced difference in the homozygous state (see genotypes CCBB′, CcBB′ and ccBB′ in Fig. 4F). Comparisons of homozygous (CC) and heterozygous (Cc) classes of *Rb3*, grouped by *Rb1* and *Rb2* allelic status, revealed no significant differences in a regression analysis (data not shown) and were therefore pooled, to create a total of 16 genotypic classes, reducing the number of missing classes in the data. Regression analysis of these data revealed that there was a significant three-way interaction between *Rb1(a*), *Rb2(b*) and *Rb3(c*) (Supplementary Table S1).

When data were grouped by *Rb1*, *Rb2* and *Rb3* (Supplementary Fig. S2) considering chromosome 13 in all three classes, *Rb3(CC*) homozygotes (Supplementary Fig. S2A), *Rb3(Cc*) heterozygotes (Supplementary Fig. S2B) and *Rb3(cc*) homozygotes (Supplementary Fig. S2C), much of the variation in root bark percentage in *Rb3(cc*) homozygotes in the absence of *Rb2(b*) was explained by the presence or absence of *Rb1(a*), with *Rb1(a*) giving the higher root bark percentage. This indicates that there is an interaction between *Rb1(a*) and *Rb3(c*) in the absence of *Rb2(b*). It is also noteworthy that regardless of the allelic status of *Rb1* in the *Rb3(cc*) homozygous class, mean levels of root bark percentage were almost the same when *Rb2(Bb*) heterozygotes were considered, indicating that the functional status of *Rb2* also affects *Rb3(cc*) expression (Supplementary Fig. S2).

A full three-locus analysis, taking into account the full allelic status of *Rb1*, *Rb2* and *Rb3* was not feasible due to the small sample size obtained once the population was further split into its representative genotypes. For this population there are up to 64 possible genotypic classes and the dataset does not contain even the 24 possible classes described in this paper; for example, the *AAbbcc* genotypic class is missing from the dataset. However, there were six individuals with *Rb1(a*) present, and both *Rb2(b*) and *Rb3(c*) in the homozygous states, i.e. *Aabbcc*. The average root bark percentage in this class was 81%, which was the highest of any genotypic class with a sample size of >2. There were three individuals where the homozygous state at all QTL loci was AABB′CC, and the average root bark percentage in this class was 58% (the genotype and root bark percentage of each individual in the M432 population are presented in Supplementary Table S3).

### Root bark percentage and rootstock-induced dwarfing

**Fig. 5.**
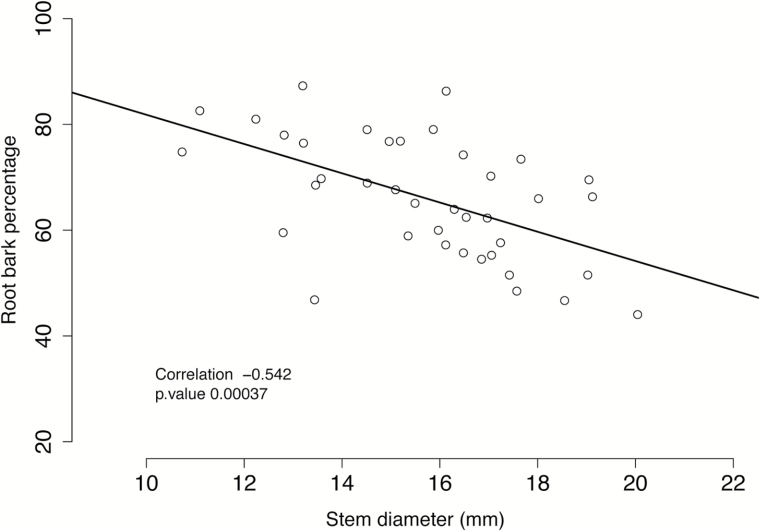
Root bark and stem diameter correlations in a grafted subset of M432 reveal a significant negative correlation (*r*=–0.567) between root bark percentage and stem diameter (Spearman rank correlation).

To test for an association between root bark percentage and rootstock-induced dwarfing in the M432 population, a small subset (*n*=38) of the M432 population that had been clonally propagated and grafted was used. Stem diameter was measured in this subset and found to be significantly negatively correlated (*r*=–0.54, *P*<0.0004) with root bark percentage (Fig. 5). Using a linear mixed model, the effects of *Rb3(c*) on stem diameter were examined. Model selection was carried out using log-transformed data as previously described, by sequentially adding terms. Presented below are two genetic models: model (a) postulated by Fazio *et al.* (2014) using their *Dw* notation, and model (b), the result of our model selection process using *Rb* notation (Supplementary Table S2):

Model (a): Stem diameter ~*Dw1*+*Dw2+Dw1×Dw2*

Model (b): Stem diameter ~*Rb1*+*Rb2*+*Rb1×Rb2+Rb2×Rb3*

Model (b) was a significantly better fit than model (a), with interactions detected between *Rb1(a*) and *Rb2(b*), and *Rb2(b*) and *Rb3(c*) (note that it was not possible to fit a three-way interaction model due to multiple missing genotypic classes in this subset of the population). Model (b) explained 84% of the variance observed in the grafted subset of the population (Supplementary Table S2), compared with 53% for model (a). Adding *Rb2×Rb3* to the model significantly increased the percentage of the variance accounted for by 31% (*P*=0.001), demonstrating that *Rb3(c*) has a non-additive effect on stem dwarfing in the presence of *Rb2(b).* This is consistent with the results obtained for root bark percentage and can therefore be considered to be analogous to a new putative QTL for rootstock-induced dwarfing *(Dw3*). Although the dataset is limited, it can be seen that the effects of *Rb3(c)/Dw3* (Supplementary Fig. S3A–C) are largely consistent with the root bark percentage data, with *Rb1(A*), *Rb2(B*) and *Rb3(C*) plants displaying the largest stem diameter (Supplementary Fig. S3A), and heterozygous *Rb3(Cc*) plants displaying a dwarfing phenotype dependent upon *Rb1* and *Rb2* (Supplementary Fig. S3B), which is enhanced in *Rb3(cc*) homozygotes (Supplementary Fig. S3C). In this case, significant differences were observed between classes heterozygous at *Rb3(Cc*) and homozygous at *Rb3(cc*), indicating that *Rb3* may have more profound effects upon stem diameter than root bark percentage.

### QTL in other common rootstocks

Three STS markers were developed that are closely linked to the root bark QTL and screened on a selection of apple rootstock material (Table 3). Interestingly, only ‘M.26’, ‘M.27’ and ‘M.9’ contained all *Rb* QTL, while other rootstocks contained either *Rb1(a*) and/or *Rb3(c*) (‘M.1’, ‘M.6’, ‘M.7’ and ‘M.16’, Table 3). The rootstocks ‘Polish 22’ and ‘Mac 9’ are both dwarfing and yet lack both *Rb1* and *Rb3*, only containing markers for *Rb2*/*Dw2*. This raises the possibility that there are other alleles or loci interacting with *Rb2/Dw2* that are absent from other ‘M.9’-derived material and that either they are unmapped in the M432 cross or recombination has occurred between markers and QTL in these accessions. This information reinforces our finding that *Rb2* is a crucial locus for highly dwarfing rootstocks.

**Table 3.** Analysis of the three *Rb* molecular markers developed in this study in a selection of apple rootstocks

		*Rb1*			*Rb2*			*Rb3*				
*Rb* alleles mapped in M432 mapping population/peak size		A	A′	a	B	B′	b	C	C′	c		
Apple rootstock	Pedigree	210	212	222	276	296	339	243	252	200	Vigour prediction	Actual vigour
M.1	Unknown	×		×	×					×	V	V
M.2	Unknown		×								V	V
M.6	Unknown	×		×						×	SD	SD
M.7	Unknown		×	×						×	SD	SD
M.9	Unknown	×		×			×		×	×	D	D
M.10	Unknown	×	×			×		×			V	V
M.11	Unknown		×				×	×	×		V	V
M.12	Unknown	×	×					×		×	V	V
M.13	Unknown		×		×	×		×	×		V	V
M.16	Unknown	×		×		×		×		×	V	VV
M.26	M.16 × M.9	×		×		×	×	×		×	D	SD
M.27	M.13 × M.9		×	×		×	×	×		×	D	DD
M.116	M.27 × M.M.106	×	×		×		×		×	×	SI	SI
M.M.106	N. Spy × M.1	×	×		×				×	×	V	V
Mac 9	M.9 open pollination	×					×		×		V	D
Northern Spy	Unknown	×	×						×	×	V	SI
Ottawa 3	Robin Crab × M.9	×	×						×		V	D
Polish 22	M.9 × Common Antonovka	×	×				×		×		V	DD

V: vigorous; SI: semi-invigorating; SD: semi-dwarfing; D: dwarfing; DD: highly dwarfing.

## Discussion

### Measurements of rootstock-induced dwarfing

Rootstock-induced dwarfing presents a ‘difficult-to-measure’ trait in rootstock studies that changes with time and can be highly influenced by many factors including soil type, climate and the interaction between rootstock and scion genotypes. Typical measurements of tree vigour are performed over several years to fully characterize the influence that a rootstock confers to a scion (Seleznyova *et al.*, 2003). This study explored a previously reported association between root bark percentage and rootstock-induced dwarfing in apple rootstocks (Beakbane and Thompson, 1947). The measurement of a primary rootstock trait confers a large advantage when phenotyping rootstocks for dwarfing ability, and root bark percentage is relatively easy to measure in cpmparison with other standard measurements of tree vigour. By measuring root bark percentage in the M432 mapping population, we were able to confirm a link between root bark percentage and rootstock-induced dwarfing, with a higher root bark percentage in the ungrafted rootstock correlating with a decrease in stem diameter when the same rootstock was grafted with a scion. Though the association between root bark percentage and rootstock-induced dwarfing was made in this population, further assessment is needed in a wider selection of germplasm to determine whether root bark percentage is a robust trait that is linked to stem dwarfing in other populations.

### Genetic interaction and model selection

This study identified QTLs for root bark percentage in the M432 mapping population, identifying three QTLs in this field grown rootstock population. Two strong QTLs, *Rb1* and *Rb2*, were identified and found to co-localize with previously determined major dwarfing loci, *Dw1* and *Dw2*, as well as QTLs for early bearing, flower density and fruit yield (Rusholme-Pilcher *et al.*, 2008; Fazio *et al.*, 2014), indicating that these chromosomal regions contain valuable rootstock traits. The positions of *Rb1* and *Rb2* concur broadly with the previously reported locations of two QTLs for stem dwarfing, *Dw1* and *Dw2*. The first of these has been reported as approximately 14 cM down linkage group 5, strongly linked to the marker CH03a09 (Rusholme-Pilcher *et al.*, 2008). This microsatellite marker is placed approximately 7.6Mb along chromosome 5 (as judged by BLASTn of sequence XM_008357860.1). From our analysis, the most significant SNP marker is approximately 14.5Mb along the physical map, though the significant QTL region encompasses the region in which *Dw1* is contained. The large interval of significance for *Rb1* in our study is due to the lack of observable recombination events on this linkage group in M27, whereby 63 out of 140 offspring showed no discernable recombination event on this linkage group. The actual number of markers used for the study of *Rb1* on linkage group 5 was 88 segregating markers. Subsequent development of a microsatellite marker to determine specific haplotypes was mapped to a position 2.4Mb along chromosome 5.

*Dw2* has been reported as lying at approximately 12Mb along the physical map of chromosome 11 flanked by CH02d08 and C13243 (Fazio *et al.*, 2014). The most significant SNP linked to *Rb2* maps to 8.4Mb along the physical map of chromosome 11, while the most significant SSR is CH03d08, which maps <1 cM away from CH02d08 in our map, confirming that *Rb2* and *Dw2* co-locate.

The third QTL, *Rb3*, was located on chromosome 13, a region that has not been previously identified in association with any important rootstock trait. *Rb3* is positioned approximately 2.2Mb along the physical map of chromosome 13 and does not co-localize with any known dwarfing locus. To our knowledge, this region on chromosome 13 has not been associated with any scion tree architecture phenotype. Through the use of a clonally propagated subset of the mapping population, a link between the effects of all three root bark loci and that of scion dwarfing was established.

The identification of eight genotypic classes (under a two-locus model) using a combination of SNP and microsatellite markers led to the clear observation that *Rb2(b*) on LG11 was absolutely required for the effect of *Rb1(a*) on LG05 to be expressed, demonstrating that there are non-additive effects of allelic combination upon the phenotype and that there is negative epistasis acting between the two loci. Furthermore, depending upon the allelic combination and allelic dosage at *Rb2*, the effect of *Rb1(a*) varied, indicating that some *Rb2* alleles were better able to compensate for the effect of *Rb1(a*) than others. The effect of *Rb3(c*) on root bark percentage appears in most situations to require *Rb1(a*) or *Rb2(b*) to be present to have a large effect (Fig. 4F and Supplementary Fig. S2). Furthermore, when *Rb3(c*) was analysed in relation to stem dwarfing, it was found to interact significantly with *Rb2(b*) to alter stem diameter (Supplementary Table S2 and Supplementary Fig. S3)—note that a full three-way analysis could not be performed in this experiement. In this study, the model of rootstock-induced dwarfing presented by Fazio *et al.* (2014), could not distinguish genotypically between the dwarfing ‘M27’ rootstock and the semi-invigorating ‘M.116’ rootstock (Fazio *et al.*, 2014). The new three-locus model developed in this study provided resolution between the two closely related genotypes and, in addition, increased the percentage variance accounted for to 84%, significantly improving the total variance accounted for by 31% (*P*=0.001).

### A central role for *Rb2/Dw2*

Previous studies have emphasized the dominant role of *Dw1(Rb1*) in rootstock-induced dwarfing (Rusholme-Pilcher *et al.*, 2008; Fazio *et al.*, 2014; Foster *et al.*, 2015), although these same studies reported cases where *Dw1* was present yet the expected phenotype did not behave as predicted. In addition, Foster and co-workers state: ‘*Dw1* has a stronger effect than *Dw2*’ and that ‘*Dw2* alone cannot induce dwarfing’, and furthermore, Fazio *et al* reported peculiarities in that ‘homozygous individuals for non-dwarfing alleles at either *Dw2* or *Dw1* invalidate the effect of the dwarfing ability of the other dwarfing locus’. Our approach in this study has enabled us to identify epistatic interactions between all three *Rb/Dw* loci, and furthermore, determine that *Rb2/Dw2* plays a significant role in rootstock-induced dwarfing. Previous reports that several rootstocks contain *Dw1* yet have vigorous phenotypes, can now be explained in the light of our findings that *Rb2/Dw2* or *Rb3/Dw3* would need to be present for the dwarfing phenotype to manifest itself. Through a marker screen of selected rootstocks, we found the dwarfing rootstocks ‘Polish 22’ and ‘Mac 9’ only contain markers for *Rb2/Dw2.* This provides support for a central role for *Dw2/Rb2*, and as previously mentioned, also raises the possibility of other interacting loci that are yet to be determined. In addition, the dwarfing rootstock ‘Ottawa 3’ does not have any of the markers linked to the three *Rb* QTLs, suggesting the *Rb* markers are unlinked to the QTLs in this genotype or that the dwarfing QTLs, previously identified by Fazio *et al.* (2014), have arisen independently, though this is unlikely with the dwarfing ‘M.9’ as a parent.

### Root bark percentage and rootstock-induced dwarfing

It cannot be stated that the amount of root bark directly affects dwarfing, as this could be the result of a pleiotropic effect of an as-yet-unidentified molecular process. However, vigour control over the scion is still observed with dwarfing rootstocks when they are used as an interstock (a stem piece of dwarfing rootstock grafted in between the rootstock and the scion to create a three-part composite tree), with the degree of dwarfing related to the length of the interstock such that a longer interstock confers a greater degree of dwarfing (Feree and Carlson, 1987). In addition, bark grafts have also been shown to reduce tree vigour, whereby a ring of bark from a tree is removed and replaced with the bark of a dwarfing rootstock (Lockard and Schneider, 1981). In both cases, the bark is implicated in the process of vigour control to the scion; in the latter case, the causative agent is reduced to a far simpler component. However, if constituents within the bark are responsible for rootstock-induced dwarfing, there is currently little information to suggest the identification of the genes responsible or the primary mechanisms underlying this phenomenon, although key hormones such as auxin, abscisic acid and cytokinins, as well as chemical compounds including phenolic acids and flavonoids, have all been implicated (Beakbane, 1956; Yadava and Lockard, 1977; Kamboj *et al.*, 1999; Van Hooijdonk *et al.*, 2010).

In woody perennial systems, there is a paucity of information on the development of the secondary vascular cambium and the cellular processes involved in the determination of cambial cell fate. The vascular cambium of trees undergoes asymmetric cell division to differentiate into secondary phloem and xylem cells. It is the cambium that is responsible for the secondary growth leading to the radial thickening of trees (Esau, 1965). The developmental and regulatory networks of vascular cambium differentiation are little understood, yet vascular cellular organization and ontogeny are central to all plant functions, playing a vital role in plant growth and development. Fundamental studies into root bark development and the genetic mechanisms underlying the determination of cambial cell fate in the developing stem and root system would provide important insights into many aspects of woody perennial development. Further research is needed to understand the underlying causes of high root bark percentage in apple rootstocks and how these cellular mechanisms may interact with the scion, including the correlation between root bark percentage and rootstock-induced dwarfing. An alternative explanation for the tight linkage between *Rb* and *Dw* loci might be that they are a consequence of linkage drag (high root bark percentage being unintentionally selected alongside rootstock-induced dwarfing) in breeding programmes. However, it is unlikely that QTLs for root bark variation and scion dwarfing would be genetically linked to all three rootstock dwarfing loci without a mechanistic explanation.

### Conclusions

This study has demonstrated a link between root bark percentage and rootstock-induced dwarfing in a rootstock mapping population. Through the identification of three QTLs for root bark percentage, a three-locus model for predicting rootstock-induced dwarfing to the scion has been developed. The rootstocks ‘M.27’ and ‘M.116’ confer different levels of vigour control towards the scion, and with the identification of 24–64 genotypic classes, we are now able to differentiate genotypically between ‘M.27’ and ‘M.116’ allowing resolution of dominance effects. In addition, we can use the information provided by the development of a three-locus model to further our understanding of rootstock-induced dwarfing by developing new mapping populations that will enable fine mapping of each QTL to identify the genes underlying it and to study the effects of individual QTL. Furthermore, this model can now be incorporated into rootstock breeding programmes to aid marker-assisted breeding strategies. This study is an important step towards the understanding of the genetic mechanism(s) underlying an important rootstock trait and has provided useful molecular markers for marker-assisted breeding.

## Supplementary data

Supplementary data are available at *JXB* online.

Figure S1. Plot of means for chromosome 5 and chromosome 11 based on the model selection procedure.

Figure S2. Plot of three-locus analysis of means for log-transformed root bark percentage.

Figure S3. Plot of three-locus analysis of means for log-transformed stem diameter.

Table S1. Model selection for root bark percentage.

Table S2. Model selection for stem diameter.

Table S3. The genotype and root bark percentage of each individual in the M432 population.

Supplementary Data
