# Supplementary material for: A new three-locus model for rootstock-induced dwarfing in apple revealed by genetic mapping of root bark percentage
Source: J Exp Bot. 2016 Jan 29;67(6):1871–81. doi: 10.1093/jxb/erw001 (PMC4783367; doi:10.1093/jxb/erw001)
Supplement: Supplementary Data [file supp_67_6_1871__index.html]

A new three-locus model for rootstock-induced dwarfing in apple revealed by genetic mapping of root bark percentage — A new three-locus model for rootstock-induced dwarfing in apple revealed by genetic mapping of root bark percentage — Supplementary Data 

# A new three-locus model for rootstock-induced dwarfing in apple revealed by genetic mapping of root bark percentage

## Supplementary Data

Data files

- supplementary\_data.pdf - Supplementary Data
