# Supplementary material for: A new three-locus model for rootstock-induced dwarfing in apple revealed by genetic mapping of root bark percentage
Source: J Exp Bot. 2016 Jan 29;67(6):1871–81. doi: 10.1093/jxb/erw001 (PMC4783367; doi:10.1093/jxb/erw001)
Supplement: Supplementary Data [file supp_erw001_supplementary_data.pdf]

A new three-locus model for rootstock-induced dwarfing in apple revealed by genetic mapping of root bark percentage.

Nicola Harrison, Richard J. Harrison, Nuria Barber-Perez, Emma Cascant-Lopez, Magdalena Cobo-Medina, Marzena Lipska, Rebeca Conde-Ruíz, Philip Brain, Peter J. Gregory and Felicidad Fernández-Fernández

SUPPLEMENTARY DATA

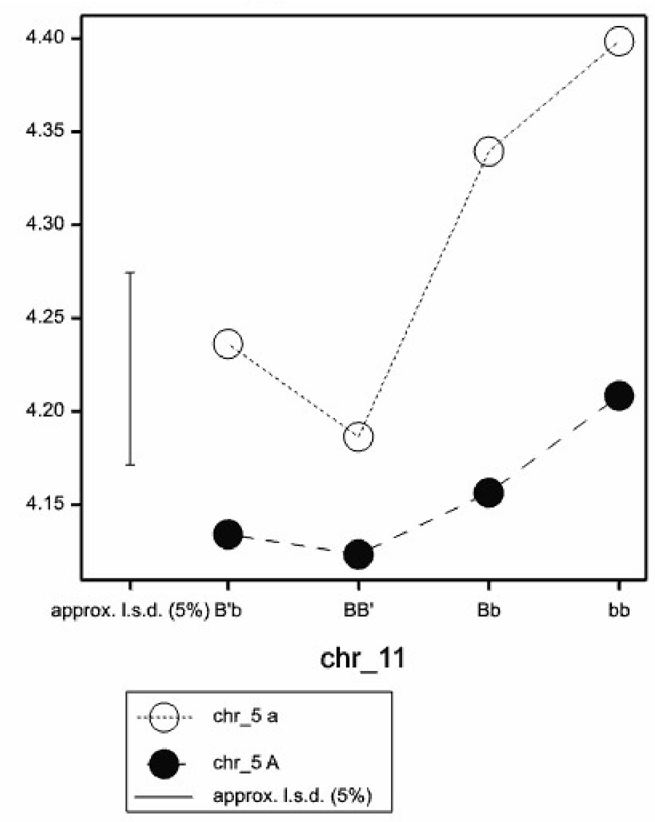

Figure S1 – Plot of means for Chromosome 5 and Chromosome 11 based on the model selection procedure.

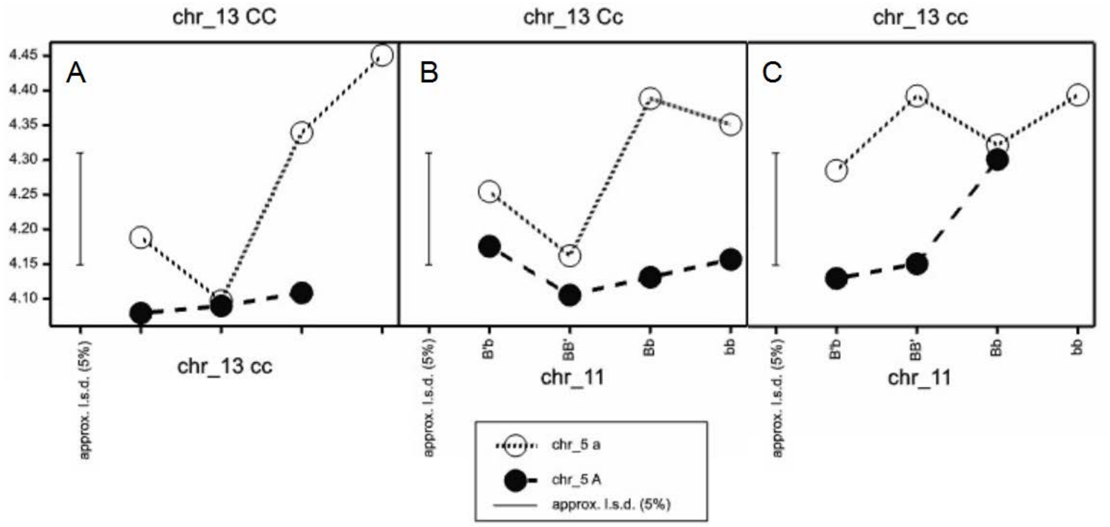

Figure S2 – Plot of three-locus analysis of means for log transformed root bark percentage. The groups of plants that are *Rb3(cc)* homozygous have overall higher mean levels of root bark percentage when *Rb1(a)* is present than when it is absent. In the absence of *Rb3(c)* root bark percentages are much lower in cases where *Rb1(a)* is present unless *Rb2(Bb or bb)* is present.

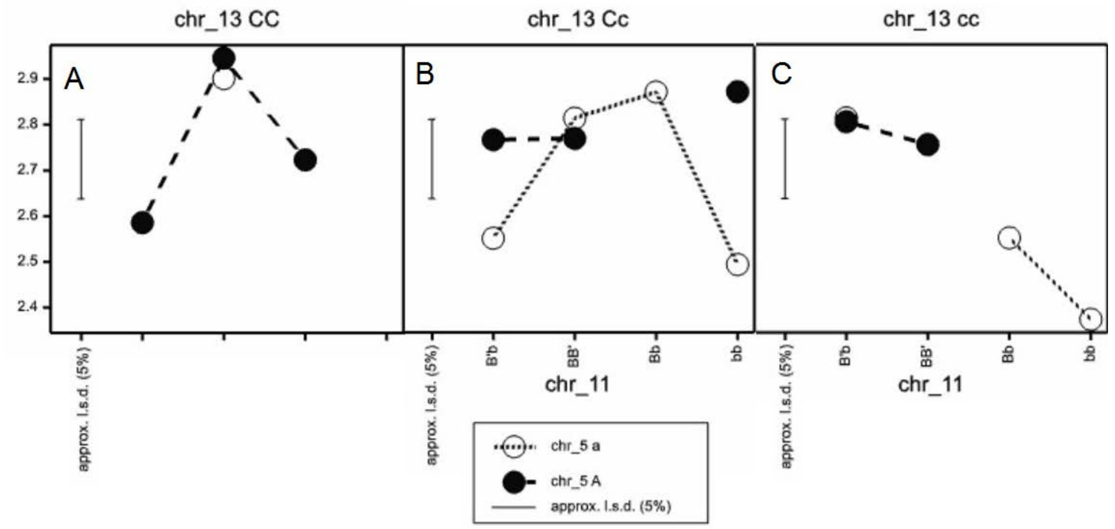

Figure S3 – Plot of three-locus analysis of means for log transformed stem diameter. The groups of plants that are *Rb3(cc)* homozygous have overall lower mean stem diameter when *Rb1(a)* is present than when it is absent. In the absence of *Rb3(c)* stem diameters are much higher in cases where *Rb1(a)* is present unless *Rb2(Bb or bb)* is present.

Table S1 – Model selection for root bark percentage. 's' in the table denotes where genotypic classes were pooled when regression analysis revealed no significant differences, thereby reducing the number of missing classes in the data.

Change	d.f.	s.s.	m.s.	v.r.	F pr.
+ chr_5	1	0.56883	0.56883	53.61	<.001
+ chr_11	3	0.26106	0.08702	8.2	<.001
+ chr_13_s	1	0.04686	0.04686	4.42	0.038
+ chr_5.chr_11	3	0.10092	0.03364	3.17	0.028
+ chr_5.chr_13_s	1	0.00008	0.00008	0.01	0.932
+ chr_11.chr_13_s	3	0.03122	0.01041	0.98	0.405
+ chr_5.chr_11.chr_13_s	2	0.08191	0.04095	3.86	0.024
Residual	94	0.9973	0.01061		
Total	108	2.08817	0.01933		

Table S2 – Model selection for stem diameter

Change	d.f.	s.s.	m.s.	v.r.	F pr.
+ chr_5	1	0.029977	0.029977	5.01	0.036
+ chr_11	3	0.148562	0.049521	8.28	<.001
+ chr_13	2	0.027331	0.013666	2.28	0.127
+ chr_5.chr_11	3	0.226195	0.075398	12.6	<.001
+ chr_5.chr_13	2	0.030507	0.015254	2.55	0.102
+ chr_11.chr_13	4	0.19676	0.04919	8.22	<.001
Residual	21	0.125663	0.005984		
Total	36	0.784994	0.021805		

Table S3 - The genotype and root bark percentage of each individual in the M432 population.

Individual	LG5_Rb1	LG11_Rb2	LG13_Rb3	M432 Field RB%
M.116	AA'	Bb	C'c	
M.27	A'a	B'b	Cc	
M432-002	--	B'b	CC'	Tree Dead
M432-003	A'A'	Bb	Cc	69.8
M432-005	A'A	bb	C'c	67.6
M432-008	Aa	B'b	CC'	68.8
M432-009	--	B'b		55.6
M432-010	Aa	B'b		90.5
M432-011	A'a	BB'	Cc	68.8
M432-013	--	B'b		78.6
M432-014	A'a	B'b	CC'	82.0
M432-015	A'A	bb	Cc	58.1
M432-016	A'A	bb	Cc	72.9
M432-017	A'a	Bb	Cc	71.8
M432-020	A'A'	B'b	Cc	Tree Dead
M432-021	Aa	B'b	cc	Tree Dead
M432-022	Aa	Bb	Cc	72.3
M432-023	A'A	Bb	CC'	60.5
M432-024	Aa	Bb	Cc	73.4
M432-025	Aa	BB'	CC'	66.7
M432-026	A'A	Bb	C'c	64.2
M432-028	A'a	Bb	C'c	75.3
M432-029	A'A'	Bb	C'c	65.4
M432-031	A'A'	B'b	C'c	56.8
M432-033	A'A	B'b	Cc	61.2
M432-034	A'A	BB'		66.3
M432-035	A'A	B'b		61.0
M432-036	Aa	Bb	cc	80.7
M432-037	A'A	B'b	CC'	Tree Dead
M432-038	A'a	Bb	C'c	64.3
M432-039	A'A'	Bb	cc	51.9
M432-040	A'a	bb	CC'	Tree Dead
M432-043	Aa	bb		Tree Dead
M432-044	A'a	BB'	cc	Tree Dead
M432-045	Aa	B'b	CC'	Tree Dead
M432-046	A'A'	bb	C'c	66.4
M432-047	Aa	BB'	CC'	68.6

M432-048	A'a	bb	CC'	Tree Dead
M432-049	A'a	B'b	cc	Tree Dead
M432-051	A'a	BB'	C'c	61.6
M432-052	A'A'	BB'	CC'	61.9
M432-053	A'a	bb	cc	84.6
M432-055	A'A	Bb	CC'	Tree Dead
M432-056	Aa	bb	C'c	Tree Dead
M432-057	Aa	bb	cc	81.6
M432-058	A'A	Bb	cc	68.7
M432-059	A'A	Bb	Cc	74.1
M432-060	A'A	bb	C'c	59.5
M432-062	A'A'	B'b	C'c	70.4
M432-064	--	B'b		Tree Dead
M432-066	A'A	bb	C'c	63.6
M432-067	Aa	bb	cc	80.7
M432-069	Aa	B'b	Cc	Tree Dead
M432-070	A'a	B'b	C'c	Tree Dead
M432-073	A'a	Bb	C'c	78.5
M432-074	A'a	bb	Cc	Tree Dead
M432-075	A'A'	BB'	cc	62.8
M432-077	A'a	B'b	C'c	Tree Dead
M432-079	--	BB'	CC'	Tree Dead
M432-081	A'A	Bb	cc	69.9
M432-083	Aa	bb	cc	Tree Dead
M432-084	A'A'	Bb	C'c	63.6
M432-085	A'A'	Bb	Cc	67.7
M432-086	A'A'	BB'	C'c	Tree Dead
M432-088	Aa	BB'	C'c	59.3
M432-089	A'A	B'b	CC'	58.0
M432-090	A'A'	B'b	CC'	Tree Dead
M432-091	--	BB'	cc	64.2
M432-092	A'A'	B'b	Cc	68.3
M432-093	A'A'	Bb	cc	70.8
M432-094	A'a	BB'	CC'	Tree Dead
M432-095	A'A	BB'	Cc	57.0
M432-096	Aa	Bb	CC'	63.7
M432-098	A'a	bb	cc	73.6
M432-100	A'a	BB'	C'c	Tree Dead
M432-101	A'A'	Bb	cc	72.7
M432-102	A'A	BB'	Cc	60.9
M432-103	A'A'	Bb	CC'	54.8

M432-104	A'A'	Bb	cc	68.2
M432-105	Aa	Bb	Cc	67.9
M432-106	A'A'	Bb	Cc	69.6
M432-107	Aa	bb	cc	83.2
M432-109	A'A'	B'b	cc	Tree Dead
M432-110	A'a	BB'	Cc	63.8
M432-112	A'A'	bb	C'c	62.4
M432-115	Aa	Bb	Cc	Tree Dead
M432-116	A'A	Bb	CC'	57.7
M432-117	A'A'	BB'	C'c	55.6
M432-118	A'A	BB'	CC'	64.0
M432-119	Aa	B'b	Cc	73.5
M432-120	A'a	bb	C'c	84.6
M432-121	A'a	BB'	CC'	51.5
M432-122	A'A'	Bb	C'c	56.4
M432-123	A'A	B'b	cc	Tree Dead
M432-124	Aa	B'b	C'c	84.4
M432-125	A'a	BB'	CC'	55.5
M432-126	A'A	Bb		62.0
M432-127	Aa	bb	CC'	82.1
M432-128	Aa	bb	CC'	89.5
M432-131	A'A'	Bb	C'c	66.7
M432-133	A'a	bb	C'c	82.7
M432-136	A'a	bb	Cc	Tree Dead
M432-139	A'A'	bb	Cc	65.9
M432-140	A'a	Bb	cc	67.8
M432-141	--	Bb	C'c	64.6
M432-142	A'A'	bb	Cc	Tree Dead
M432-145	--	BB'	Cc	Tree Dead
M432-146	A'a	BB'	CC'	54.1
M432-147	A'A'	bb	C'c	56.7
M432-148	--	bb		64.0
M432-149	Aa	bb	cc	81.3
M432-150	A'A	B'b	Cc	50.1
M432-151	A'a	Bb	Cc	66.2
M432-152	A'A	B'b	CC'	54.7
M432-155	A'A'	B'b	Cc	81.5
M432-156	A'a	bb		75.9
M432-157	Aa	bb	cc	85.1
M432-158	A'A	Bb	Cc	56.6
M432-159	Aa	bb	C'c	71.9

M432-160	A'A'	bb	Cc	69.3
M432-161	--	bb	CC'	Tree Dead
M432-162	A'A	bb	C'c	61.5
M432-163	A'a	B'b	CC'	71.7
M432-165	A'A'	bb	Cc	60.1
M432-166	A'A'	Bb	cc	53.9
M432-167	A'A'	B'b	cc	84.2
M432-168	Aa	B'b	Cc	65.1
M432-169	Aa	B'b	cc	85.2
M432-170	Aa	Bb	Cc	58.1
M432-171	Aa	BB'	cc	69.8
M432-172	A'A'	bb	Cc	63.8
M432-173	A'A'	BB'	CC'	60.1
M432-175	Aa	BB'		52.4
M432-176	A'A'	bb	Cc	Tree Dead
M432-177	A'A'	BB'	CC'	62.9
M432-178	--	bb	Cc	86.9
M432-179	A'a	bb	C'c	75.0
M432-180	--	B'b	C'c	61.1
M432-183	A'a	BB'	cc	80.9
M432-185	Aa	B'b		66.4
M432-186	A'A'	Bb	CC'	65.9
M432-188	A'A'	B'b	cc	64.7
